# Factor Analysis of the Short Form Cohen Mansfield Agitation Inventory and Measurement Invariance by Gender

**DOI:** 10.1093/geroni/igaa057.1214

**Published:** 2020-12-16

**Authors:** Anju Paudel, Barbara Resnick, Elizabeth Galik

**Affiliations:** 1 University of Maryland, Glen Burnie, Maryland, United States; 2 University of Maryland School of Nursing, Baltimore, Maryland, United States; 3 University of Maryland, Baltimore, Maryland, United States

## Abstract

Background: The Cohen-Mansfield Agitation Inventory (CMAI), available in both long and short versions, is a widely used measure to assess and evaluate agitation among older adults. There has been less psychometric testing of the short-form CMAI particularly with regard to the factor structure of this shorter measure. Purpose: The purpose of this study was to test the internal consistency, reliability and validity of short-form CMAI in a sample of nursing home residents and examine if it is invariant across gender. Specifically, it was hypothesized that consistent with the long form CMAI, the short-form CMAI would have three factors with acceptable internal consistency and item reliability. In addition, it was hypothesized that there would be no difference in factor structure and factor means across gender. Methods: This study utilized baseline data from a randomized trial including 553 residents from 55 nursing homes. Data was analyzed using structural equation modeling. Results: Confirmatory factory analysis supported the three-factor structure of short-form CMAI including aggressive (α= 0.794), physically non-aggressive (α= 0.617), and verbally agitated (α= 0.718) behaviors; three items loading on physically non-aggressive behaviors had R2 close to 0.3 suggesting low reliability. Invariance testing confirmed that the shortened measure is invariant across gender. Conclusions: Short-form CMAI is a valid and reliable scale to assess agitation and gender differences in agitation in nursing home population. However, it could benefit from rewording the items with low reliability or, merging them with other similar items. Future work could also consider a four-factor structure for this shortened measure.

